# Abnormal networks of immune response-related molecules in bone marrow cells from patients with rheumatoid arthritis as revealed by DNA microarray analysis

**DOI:** 10.1186/ar3364

**Published:** 2011-06-16

**Authors:** Hooi-Ming Lee, Hidehiko Sugino, Chieko Aoki, Yasunori Shimaoka, Ryuji Suzuki, Kensuke Ochi, Takahiro Ochi, Norihiro Nishimoto

**Affiliations:** 1 Graduate School of Frontier Biosciences, Osaka University, 1-3 Yamada-Oka, Suita, Osaka 565-0871, Japan; 2 Laboratory of Immune Regulation, Wakayama Medical University, 105 Saito Bio Innovation Center, 7-7-20 Saito-Asagi, Ibaraki, Osaka 567-0085, Japan; 3 Yukioka Hospital, 2-2-3 Ukita, Kita-ku, Osaka 530-0021, Japan; 4 Clinical Research Center for Allergy and Rheumatology, Sagamihara National Hospital, National Hospital Organization, 18-1 Sakuradai, Sagamihara, Kanagawa 252-0392, Japan; 5 Kawasaki Municipal Kawasaki Hospital, 12-1 Shinkawa-dori, Kawasaki-ku, Kawasaki, Kanagawa 210-0013, Japan; 6 Osaka Police Hospital, 10-31 Kitayama-chou, Tennoji-ku, Osaka 543-0035, Japan

## Abstract

**Introduction:**

Rheumatoid arthritis (RA) is a systemic autoimmune disease characterized by chronic synovitis that progresses to destruction of cartilage and bone. Bone marrow (BM) cells have been shown to contribute to this pathogenesis. In this study, we compared differentially expressed molecules in BM cells from RA and osteoarthritis (OA) patients and analyzed abnormal regulatory networks to identify the role of BM cells in RA.

**Methods:**

Gene expression profiles (GEPs) in BM-derived mononuclear cells from 9 RA and 10 OA patients were obtained by DNA microarray. Up- and down-regulated genes were identified by comparing the GEPs from the two patient groups. Bioinformatics was performed by Expression Analysis Systemic Explorer (EASE) 2.0 based on gene ontology, followed by network pathway analysis with Ingenuity Pathways Analysis (IPA) 7.5.

**Results:**

The BM mononuclear cells showed 764 up-regulated and 1,910 down-regulated genes in RA patients relative to the OA group. EASE revealed that the gene category response to external stimulus, which included the gene category immune response, was overrepresented by the up-regulated genes. So too were the gene categories signal transduction and phosphate metabolism. Down-regulated genes were dominantly classified in three gene categories: cell proliferation, which included mitotic cell cycle, DNA replication and chromosome cycle, and DNA metabolism. Most genes in these categories overlapped with each other. IPA analysis showed that the up-regulated genes in immune response were highly relevant to the antigen presentation pathway and to interferon signaling. The major histocompatibility complex (MHC) class I molecules, human leukocyte antigen (HLA)-E, HLA-F, and HLA-G, tapasin (TAP) and TAP binding protein, both of which are involved in peptide antigen binding and presentation via MHC class I molecules, are depicted in the immune response molecule networks. Interferon gamma and interleukin 8 were overexpressed and found to play central roles in these networks.

**Conclusions:**

Abnormal regulatory networks in the immune response and cell cycle categories were identified in BM mononuclear cells from RA patients, indicating that the BM is pathologically involved in RA.

## Introduction

Rheumatoid arthritis (RA) is a systemic autoimmune disease characterized by chronic synovitis that is often pathogenic and destructive to articular cartilage and bone. To understand the complex pathogenesis and heterogeneous manifestations of autoimmune diseases including RA, DNA microarray has emerged as a powerful tool [1-4]. We have shown in studies investigating the pathogenesis of juvenile idiopathic arthritis (JIA) and systemic lupus erythematosus (SLE) that DNA microarray can be even more effective when combined with bioinformatics techniques such as gene ontology databases and network pathway analysis software [5,6].

In RA pathology, fibroblast-like synoviocyte (FLS) has been shown to play an essential role in the chronic inflammation of RA joints [7]. Therefore, a number of gene expression profiling studies have focused on synovial tissue or FLS to understand the aberrant biological pathways that contribute to the pathogenesis of RA [1]. Others have focused on peripheral blood mononuclear cells (PBMC) from RA patients, either by comparing them with PBMC from healthy individuals or from patients with other autoimmune diseases [1,3]. Of greater interest to us is the accumulating evidence suggesting that abnormalities in the bone marrow (BM) have a significant role in RA inflammation [2,8,9].

The BM contains three types of stem cells: hematopoietic stem cells (HSCs), which produce all the mature blood lineages for leukocytes, erythrocytes, and platelets; mesenchymal stem cells, which can differentiate into osteoblasts, chondrocytes, and adipocytes; and endothelial stem cells. The proliferations and differentiations of these heterogeneous cell populations are dependent on the BM microenvironment and are regulated by highly sophisticated networks, either through cell-cell interactions or cytokine networks. Indeed, a remarkable elevation in IL6 and IL8 levels in the BM serum from RA patients has been reported to relate to the synovial proliferation seen in multiple joints [10]. Therefore, BM cells may be where the pathogenesis of RA originates, making the study of their abnormal regulatory networks very important.

In this study, we identify aberrant regulatory networks in BM cells from RA patients by analyzing differentially expressed genes based on their gene expression profiles with those of osteoarthritis (OA) patients. OA patients were chosen because the OA pathology is relatively well understood and the BM cells from these patients are far more readily available than those from healthy subjects.

## Materials and methods

### Human subjects and ethical considerations

Nine patients (all women, median age 73 years, range 41 to 77 years) with RA satisfying the 1987 revised diagnostic criteria of the American College of Rheumatology [11] and 10 patients with OA (all women, median age 69 years, range 39 to 90 years) fulfilling American College of Rheumatology criteria for hip or knee OA [12] were enrolled in the present study after obtaining their written informed consent. The study was reviewed and approved by the Ethical Committee of Wakayama Medical University. BM fluid was intraoperatively obtained from the nine RA patients and the 10 OA patients while undergoing joint arthroplasty. Detailed patient characteristics, medication usage, including disease-modifying agents and steroids, and laboratory testing, including rheumatoid factor (RF) and C-reactive protein (CRP), of the nine RA patients are shown in Table 1. None of the 10 OA patients received steroids or disease-modifying antirheumatic drugs (DMARDs).

**Table 1:** Demographic and rheumatoid arthritis disease characteristics

Patient	RA disease	RF	CRP	steroid	DMARDs
**no**.	duration (years)	(Unit/mL)	(mg/dL)	(mg/day)	
1	21	172	3.3	10	MTX 6 mg/week
2	24	99	2.0	5	Bucillamine 100 mg/day, MTX 4 mg/week
3	40	507	3.3	0	Actarit 200 mg/day
4	45	49	0.3	0	
5	9	324	2.6	4	
6	16	13	1.4	2.5	Bucillamine 100 mg/day
7	2	242	4.4	7.5	Salazosulfapyridine 1000 mg/day, MTX 6 mg/week
8	24	98	0.9	0	MTX 4 mg/week
9	17	40	2.9	5	MTX 6 mg/week

CRP, C-reactive protein; DMARDs, disease-modifying antirheumatic drugs; MTX, methotrexate;

RA, rheumatoid arthritis; RF, rheumatoid factor.

### GeneChip microarray and data analysis

Patient BM were collected and kept at 4°C. All BM-derived mononuclear cells (BMMC) were isolated by using Ficoll-Paque™ Plus (GE Healthcare Biosciences, Tokyo, Japan) gradient centrifugation according to the manufacturer's recommendations. Total RNA from the BMMCs was extracted by using the RNeasy Mini Kit (Qiagen, Tokyo, Japan). A 3 μg sample of total RNA was used for DNA microarray analysis by using GeneChip Human Genome U133 Plus 2.0 Array (Affymetrix, Santa Clara, CA, USA). Signal values were obtained according to the manufacturer's instructions and normalized by eliminating the highest and lowest 2% of the data, respectively. Only data with present or marginal detection calls were selected for further analysis. Microarray data have been deposited in NCBIs Gene Expression Omnibus (GEO) and are accessible through GEO series accession number [GSE27390].

### Gene ontology and network pathway analysis

Genes were identified as differentially expressed if their mean signal values were at least 50% different between the RA and OA groups. These genes were functionally categorized using Expression Analysis Systematic Explorer (EASE) version 2.0 bioinformatics software [13]. Interactions among the differentially expressed genes in each gene category were investigated by using Ingenuity Pathway Analysis (IPA) version 7.5 [14]. Networks generated by less than 10 uploaded genes were excluded from the analysis.

### Statistical analysis

The false-discovery rate was used to determine statistically significant differences in the mRNA expression levels between the RA and OA groups. The criterion for the statistical significance was q < 0.001.

## Results

### Gene ontology analysis for differentially expressed genes in RA and OA patients

DNA microarray analysis revealed that 2,674 genes were differentially expressed in BMMC from patients with RA compared with those from patients with OA: 764 out of the 2,674 genes were up-regulated and the remaining 1,910 genes were down-regulated.

To identify any aberrant biological function in the BMMC of RA patients, EASE based on the Gene Ontology (GO) database, which can classify large gene lists into functionally related gene groups and rank their importance, was performed. EASE classified the gene categories into three GO systems: biological process, cellular component, and molecular function. Up-regulated and down-regulated genes for the GO system biological process based on EASE are shown in Tables 2 and 3, respectively. The EASE score, which is a modified Fisher's exact test, represents the probability that an over-representation of a certain gene category occurs by chance. Based on common genes, the gene categories were further divided into subsets. Each subset of a gene category was then ordered hierarchically based on the gene list. Identical gene lists are listed as one gene category. The parameter list refers to the total number of up- or down-regulated genes annotated in the GO system (not shown). There were 348 genes in the list for the 764 up-regulated genes and 733 genes in the list for the 1,910 down-regulated genes. List hits shows the number of up- or down-regulated genes that belong to a respective gene category. The parameter population reports all genes annotated in the GO system (not shown). The total number of genes in the population for biological process is 10,937. Population hits shows the number of genes that belong to a respective gene category in the system.

**Table 2:** Top 15 deviated gene categories of overexpressed genes in rheumatoid arthritis bone marrow compared with osteoarthritis bone marrow

Gene category	List hits	Population hits	EASE score
(GO biological process)	(Total = 348)	(Total = 10,937)	
response to external stimulus	67	1263	2.52E-05
response to biotic stimulus	61	821	6.47E-10
defense response	57	756	1.62E-09
immune response	56	682	9.89E-11
response to pest/pathogen/parasite	27	444	1.92E-03
antigen processing, endogenous antigen via MHC class I	4	12	5.63E-03
response to stress	37	784	1.57E-02
signal transduction	97	2196	3.43E-04
intracellular signaling cascade	47	761	1.62E-05
protein kinase cascade	12	155	1.04E-02
activation of MAPK	4	16	1.30E-02
phosphate metabolism	33	662	1.13E-02
phosphorylation	28	524	8.90E-03
protein amino acid phosphorylation	26	478	9.74E-03
RNA splicing, via transesterification reactions	8	83	1.61E-02

EASE, Expression Analysis Systematic Explorer software, Version 2.0; Gene Ontology database; GO, gene ontology; MAPK, mitogen activated protein kinase; MHC, major histocompatibility complex.

**Table 3:** Top 15 deviated functional categories of underexpressed genes in rheumatoid arthritis bone marrow compared with osteoarthritis bone marrow

Gene category	List hits	Population hits	EASE score
(GO biological process)	(Total = 733)	(Total = 10,937)	
cell proliferation	139	1036	4.05E-16
cell cycle	127	690	1.06E-26
mitotic cell cycle	97	329	2.78E-37
M phase	48	157	3.98E-19
nuclear division	47	151	4.40E-19
mitosis	44	121	7.91E-21
regulation of mitosis	11	26	3.37E-06
regulation of cell cycle	64	384	1.83E-11
cell cycle checkpoint	15	35	1.98E-08
cytokinesis	26	95	1.83E-09
DNA replication and chromosome cycle	52	186	8.28E-19
DNA replication	40	146	2.96E-14
DNA dependent DNA replication	23	75	1.86E-09
DNA metabolism	85	517	1.02E-14
nucleocytoplasmic transport	21	97	5.06E-06

EASE, Expression Analysis Systematic Explorer software, Version 2.0; Gene Ontology database; GO, gene ontology.

EASE of the up-regulated genes identified four major gene categories: response to external stimulus, signal transduction, phosphate metabolism, and RNA splicing, via transesterification reactions (Table 2). Based on EASE scores, response to biotic stimulus (EASE score: 6.47E-10), defense response (1.62E-09), and immune response (9.89E-11) were the three most significant gene categories that corresponded with response to external stimulus. Fifty-six of the 67 genes in response to external stimulus belonged to immune response, which had the lowest EASE score (9.89E-11). The genes in signal transduction corresponded to intracellular signaling cascade, protein kinase cascade, and activation of mitogen-activated phosphate kinase (MAPK). Twenty-six of the 33 genes in phosphate metabolism belonged to protein amino acid phosphorylation. Finally, there were eight genes in RNA splicing, via transesterification reactions.

EASE for the down-regulated genes identified three major gene categories: cell proliferation, DNA replication and chromosome cycle, and DNA metabolism (Table 3). The down-regulated genes were predominantly classified into cell cycle (EASE score: 1.06E-26), mitotic cell cycle (2.78E-37), M phase (3.98E-19), nuclear division (4.40E-19), and mitosis (7.91E-21). These gene categories were arranged hierarchically in cell proliferation, which contains 139 genes. Genes related to regulation of cell cycle also belonged to cell proliferation with significant probability (1.83E-11). Most of the genes in the three major gene categories overlapped.

### Up-regulated genes in the category immune response and their corresponding network pathway analysis

The gene category immune response for up-regulated genes and mitotic cell cycle for down-regulated genes had the lowest EASE scores, respectively. The relations among the 56 up-regulated genes and the 97 down-regulated genes in these two gene categories were further analyzed by IPA.

**Figure 1:**
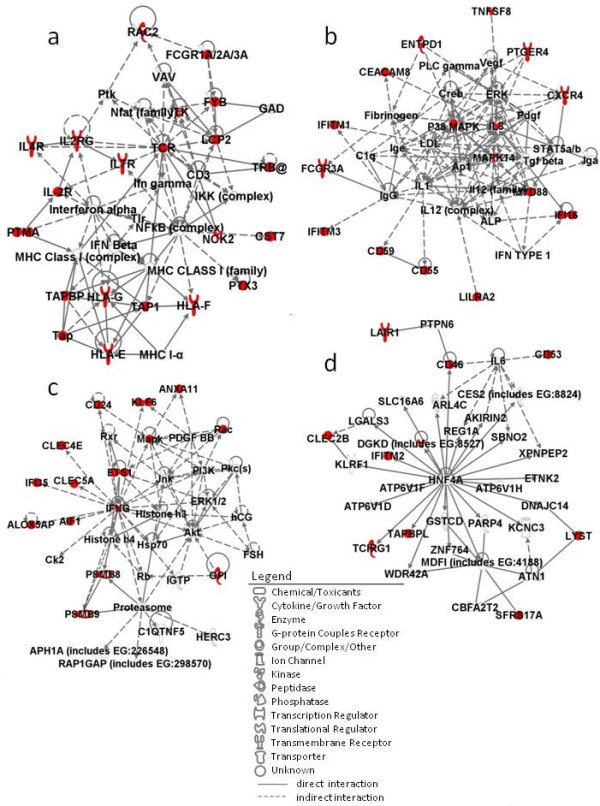
**Network pathway analysis of up-regulated genes in the gene category immune response**. (a to d) Four different networks constructed by the 56 up-regulated genes. Genes and gene products are represented as individual nodes whose shapes represent the functional class of the gene products. The biologic relation between two nodes is represented as an edge (line). All edges are supported by at least one reference from the Ingenuity Pathways Knowledge Base (IPKB). Genes in colored nodes are overexpressed. Genes in uncolored nodes are not, but are depicted by the computationally generated networks on the basis of evidence stored in the IPKB indicating a strong biologic relevance to that network.

IPA analysis revealed that the up-regulated genes in immune response were highly relevant to the antigen presentation pathway and to interferon (IFN) signaling. There were four networks represented by the 56 up-regulated genes (Figure 1). The first network (Figure 1a) has a T-cell receptor (TCR), IFN-alpha, and nuclear factor kappa B (NFkB) complex at its center. Several cytokine receptors such as IL2 receptor (IL2R), IL4R, and IL7R are depicted in this network. A cluster of human leukocyte antigens (HLA), HLA-E, HLA-F, and HLA-G, which are all major histocompatibility complex (MHC) class I molecules, tapasin (TAP), and TAP binding protein (TAPBP) are also represented in this network. The second network (Figure 1b) has the p38 MAPK complex, MAPK14, IL8, and myeloid differentiation primary response gene 88 (MyD88) at its center. Proinflammatory cytokines such as IL1 and IL12 (complex), and type I IFN are also found in the network although neither the expression of IFNα nor IFNβ are significantly up-regulated. FCγR3A, CXCR4, and three IFN-inducible (IFI) molecules, IFITM1, IFITM3, and IFI16 are found up-regulated and included in the network. The third network is found to have IFNγ play a central role (Figure 1c). The proteasomes PSMB8 and PSMB9, two C-type lectin family molecules, CLEC5A and CLEC4E, IFI35, and arachidonate 5-lipoxygenase-activating protein (ALOX5AP) are depicted in this network. The fourth and final network has hepatocyte nuclear factor (HNF) 4A at its center. HNF4A is a nuclear transcription factor that binds DNA as a homodimer. Besides the regulation of transcription, it is also involved in the regulation of the lipid metabolic process, blood coagulation, and negative regulation of cell growth. The up-regulated molecules CD46 and CD53 are also found in this network, whereas IL6 is found to be involved in its regulation.

### Down-regulated genes in the category mitotic cell cycle and their corresponding network pathway analysis

**Figure 2:**
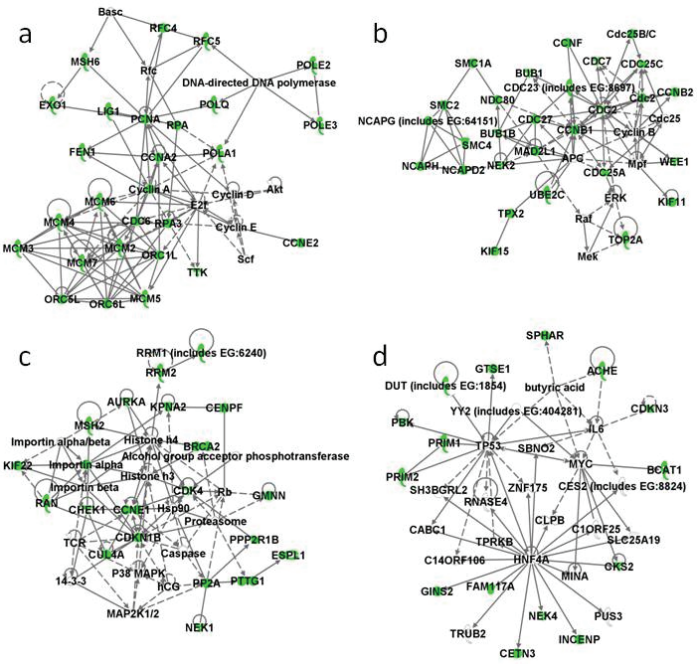
**Network pathway analysis of down-regulated genes in the gene category mitotic cell cycle**. (a to d) Four separated networks constructed by the down-regulated genes.

IPA found down-regulated molecules to significantly affect the role of polo-like kinase in mitosis, the role of CHK protein in cell cycle checkpoint control, and affect pyrimidine metabolism, and ataxia telangiectasia mutated (ATM) signaling. There were four networks constructed by the 97 down-regulated genes in the mitotic cell cycle (Figure 2). Several cyclins (CCN), cell division cycle (CDC)-related molecules, and cyclin-dependent kinase (CDK)-related molecules played central roles in the first three networks. CCNA2, CCNE2, CDC6, a group of polymerase (POL) molecules including POLA1, POLE2, POLE3, and POLQ, six mini-chromosome maintenance (MCM) complex component genes, and three origin recognition complex (ORC) subunit genes are depicted in the first network (Figure 2a). CCNB1 and CDC2 are found at the center of the second network (Figure 2b). CDC27, CDC25A, CCNF, WEE1, topoisomerase II α (TOP2A), three structural maintenance of chromosome (SMC)-related molecules, and two kinesin family member (KIF)-related molecules are also represented in the second network. CCNE1, CDK inhibitor 1B, and histones are involved in the third regulatory network (Figure 2c). In the last network, although the expression of IL6, TP53, and HNF4A were not differentially expressed, they are all included in this network and play key roles in its regulation (Figure 2d).

## Discussion

It is commonly known that autoimmunity plays a pivotal role in the pathology of RA. However, the exact etiology and pathogenesis are poorly understood. Our work, comparing the gene expression profiles of BMMC between RA patients and OA patients by microarray technology and gene ontology analysis, found abnormal immune responses in BMMC. This agrees with accumulating evidence indicating that abnormalities in BM cells may contribute to the pathogenesis of RA [9]. To our knowledge, ours is the first report to combine DNA microarray with bioinformatics for describing gene expression profiles from RA BM cells and for revealing abnormal networks involving immune response- and cell cycle-related molecules in those cells.

Several reports have shown that peripheral blood from SLE patients has remarkably homogenous gene expression patterns and an overexpression of IFI genes [6,15-17]. The IFN signaling pathway is thought to play an important role in the pathogenesis of SLE. There is also one report of genomically profiled peripheral blood cells from 35 RA patients and 15 healthy controls that found a type I IFN signature in a subpopulation of RA patients [3]. Here, we show that the IFN signaling pathway elevates in the BM cellular network pathway of RA patients similar to that in the peripheral blood of SLE patients, although to a lesser degree. The different IFN effects on RA and SLE may be because cytokines are pleiotropic in their biological activities and that they interact with each other in highly sophisticated networks. Along these lines, the effects of IFNβ treatment on arthritis were reviewed several years ago. An open, phase I study conducted on 12 patients with active RA and another pilot study performed on six children with juvenile RA have both shown that IFNβ treatment is in general well tolerated and leads to improvement [18]. However, two other case reports claim RA can develop after the onset of IFNβ treatment in patients with multiple sclerosis [18]. These suggest IFNβ therapy cannot be used universally to combat the development of arthritis.

Meanwhile, our finding that the MHC class I molecules HLA-E, HLA-F, and HLA-G, TAP, and TAPBP were all overexpressed in the BM cells of RA patients is also novel. All these genes relate to the antigen presentation pathway. For example, up-regulation of HLA-E is considered a potential marker for cancer. Additionally, its expression can confer resistance to NK cell-mediated lysis [19,20]. HLA-F has been recently reported to be a surface marker for activated lymphocytes [21], while HLA-G has its highest expression during pregnancy and is thought to play a key role in modulating immune tolerance [22]. There is a recently published study by Prigione et al. that reports a lower concentration of soluble HLA-G in sera may predispose to JIA and soluble HLA-E concentration in synovial fluid correlated with the number of affected joints [23]. Nevertheless, the functions of these molecules in autoimmunity are still unclear and debated. In addition, we found TCR, IFNα, NFkB, p38MAPK, IL8, MyD88, and IFNγ play central roles in the immunoregulatory networks of BMMC in RA. Except for NFkB, we found all these genes to be overexpressed. MyD88, the Toll/IL-1 receptor (TIR)-containing adaptor, is used by almost all Toll-like receptors (TLRs) to activate a common signaling pathway that results in the activation of NFkB to express cytokine genes involved in inflammation, as well as IFN-inducible genes [24,25]. It is possible the up-regulation of MyD88 has a significant role on the aberrant immune response network seen in BMMC from RA patients. However, our data do not show a complementary up-regulation of TLRs, nor do they confirm that the up-regulation of MyD88 was caused by TLRs. It is interesting that Nagata reported up-regulation of IFN-inducible genes in DNase II-deficient mice, which develop a chronic polyarthritis resembling human RA, and they further found no involvement of a TLR system in the IFNβ gene activation in DNase II^-/- ^embryos [26]. Kawane et al. also recently showed that when BM cells from the DNase II-deficient mice were transferred to the wild-type mice, they developed arthritis [27]. Although the mechanisms of arthritis pathogenesis may be different between mice and humans, these mouse-model data do provide supportive evidence to our report.

Another interesting observation is that underexpressed genes were dominantly related to cell cycle and DNA metabolism. We are the first to report the suppression of cell cycle and DNA metabolism in BM cells from RA patients. Initially, there appear to be several possible mechanisms that can explain this result. One is a therapeutic effect caused by MTX, as MTX acts by inhibiting the metabolism of folic acid, which is needed for the *de novo *synthesis of the nucleoside thymidine required for DNA synthesis. However, subsequent analysis showed MTX treatment does not correlate with the down-regulated gene expressions (data not shown). Alternatively, we considered the fact the BMMC samples in this study were isolated by using Ficoll-Paque, which may cause nucleated erythroblasts to be miscible in mononuclear cell proportions and thus affect cell cycle. Finally, a high concentration of serum IL6 in BM has been reported in RA patients [10]. This is important because IL6 induces the secretion of hepcidin, a humoral factor regulating intestinal iron absorption and iron storage in microphages [28,29]. Hepcidin can contribute to low serum iron levels if up-regulated, which can then suppress erythroblast differentiation and proliferation in BM, as iron is a requisite element for this process. Furthermore, Colmegna et al. reported a defective proliferative capacity by peripheral blood hematopoietic progenitor cells from RA patients [30]. They further showed that ATM deficiency in RA patients disrupts DNA repair and renders T cells sensitive to apoptosis [31]. Together with their results and our finding that the ATM signaling pathway is repressed in the immunoregulatory networks of BMMC, we suggest that in RA patients, impairments in their immune response cells originally occur in the BM. However, more work is needed on a number of issues including why cell cycle and DNA metabolism were suppressed in the BM, how this suppression relates to RA, and whether defective BM cells relate to activated-immune responses in RA patients.

According to our unpublished data, the genes expressed in the peripheral blood cells of RA patients that correspond to cell cycle and DNA metabolism were not down-regulated as observed in BM cells, but the down-regulation for those in RNA metabolism- or translation-related genes were found. As all mature blood lineages in peripheral blood are produced from HSCs in the BM, the s abnormality in immune response and suppression of cell cycle in BM may contribute to the pathogenesis of RA.

## Conclusions

BM cells from RA patients had abnormal functional networks in immune response and cell cycle when compared with the BM cells from OA patients. Our results suggest that the overexpression of genes that take part in the antigen presentation pathway and IFN signaling contribute to the pathogenesis of RA. Our results also suggest that the underexpression of genes relating to cell cycle in the BM may be a potential pathogenic factor for RA.

## Abbreviations

ATM: ataxia telangiectasia mutated; BM: bone marrow; BMMC: BM-derived mononuclear cells; CCN: cyclin; CDC: cell division cycle; CDK: cyclin-dependent kinase; CRP: C-reactive protein; DMARDs: disease-modifying antirheumatic drugs; EASE: expression analysis systematic explorer; FLS: fibroblast-like synoviocyte; GEO: Gene Expression Omnibus; GO: gene ontology; HLA: human leukocyte antigen; HNF: hepatocyte nuclear factor; HSCs: hematopoietic stem cells; IFI: IFN-inducible; IFN: interferon; IL: interleukin; IPA: ingenuity pathway analysis; JIA: juvenile idiopathic arthritis; MAPK: mitogen-activated protein kinase; MHC: major histocompatibility complex; MyD88: myeloid differentiation primary response gene 88; NFκB: nuclear factor of kappa light polypeptide; OA: osteoarthritis; PBMC: peripheral blood mononuclear cells; POL: polymerase; RA: rheumatoid arthritis; RF: rheumatoid factor; SLE: systemic lupus erythematosus; TAP: tapasin; TAPBP: TAP binding protein; TCR: T cell receptor; TLRs: Toll-like receptors.

## Competing interests

The authors declare that they have no competing interests.

## Authors' contributions

H-ML performed the data and statistical analysis, and drafted and revised the manuscript. HS and CA assisted with the acquisition of data and analysis. RS performed mRNA expression analysis with microarrays. YS and KO treated and recruited the patients for this study, and analyzed the clinical data of the patients. TO and NN made substantial contributions to the conception and design of the experiments, and analysis and interpretation of the data. All authors read and approved the final manuscript.
